# Re-reply to Sabbagh et al.

**DOI:** 10.1038/s41431-025-01806-z

**Published:** 2025-02-20

**Authors:** Diane Van Opstal, Brigitte H. W. Faas

**Affiliations:** 1 Department of Clinical Genetics, Erasmus MC, University Medical Center, Rotterdam, The Netherlands; 2 Department of Human Genetics, Radboud university medical center, Nijmegen, The Netherlands

**Keywords:** Genetic testing, Reproductive disorders

## To the Editor:

We thank you for giving us the opportunity to respond to the reply of Sabbagh et al. [1] on our correspondence [2], since there seems to be a misunderstanding concerning the methodology that we propose. When we advised to analyse DNA from the mesenchymal core (MC) of chorionic villi (CV) solely or separately, we meant the *uncultured* and not the *cultured* MC, as Sabbagh et al. are suggesting in their reply. Since their reply primarily focuses on the limitations of using MC cultures, we urgently feel the need to take away this misconception.

First of all, like Sabbagh et al., we neither want to delay a prenatal diagnosis by first culturing the CV. The cytotrophoblast (CTB) and MC can easily be dissociated with trypsin, as originally described by Smidt-Jensen et al. [3]. After an additional collagenase treatment of the MC, DNA isolation for (rapid) molecular (cytogenetic) testing can immediately be carried out on the cells obtained with this procedure, and this DNA is sufficient and suitable for all types of molecular tests. Thus, we advocate to use DNA from the *uncultured* MC for the initial requested tests, but because of the preciousness of the material, we always recommend to establish a back-up culture from the MC, for situations in which more DNA or cells are needed.

Secondly, Sabbagh et al. also advocate the use of undissociated villi to enable the detection of CPM type 1, which is associated with an increased risk on fetal growth restriction and pregnancies with CPM type I might benefit from close ultrasound monitoring [4]. Also for this purpose, we would strongly advise to analyse uncultured CTB and MC separately. Since in CPM type 1 the MC is cytogenetically normal, using DNA from a mixture of both cell layers may result in dilution of the chromosome aberration present in the CTB, and potentially even mask the presence of CPM type 1. On the other hand, CPM type I can be regarded as a risk factor for growth restriction. As prenatal diagnosis not primarily deals with risk factors, not all laboratories investigate cells from both the CTB and MC, a strategy that can also be driven by economic factors. Most laboratories restrict the analysis to the MC, that better matches the foetus because of their common embryonic origin (inner cell mass).

Thirdly, detection of CPM in general may indeed uncover foetal uniparental disomy (UPD) caused by trisomic rescue. However, the majority of foetal UPD is related to CPM type 3, in which both CTB and MC are affected due to the meiotic origin of the trisomy [5]. The case of Grati et al. [6] referred to by the authors, is an exceptional case of trisomy 14 mosaicism in CTB and normal MC cultures. However, since only eight metaphases could be analysed from the MC, excluding a mosaic of 32% with 95% confidence [7], this does not exclude a type 3 CPM, as stated by Grati et al. themselves in the paper. Moreover, most laboratories use genomic microarrays nowadays, preferably SNP arrays, allowing low level mosaicism and foetal UPD detection simultaneously.

Finally, if a chromosome aberration is restricted to the CTB and the uncultured MC is normal, a chromosomally normal foetus is to be expected, based on a large experience with CV cytogenetics [8]. Indeed, there have been a few cases of so-called true foetal mosaicism (TFM) type IV in literature, with an abnormal CTB, normal MC and an abnormal foetus, but these cases are based on classical karyotyping of a few metaphases of cultured MC, as in the case of trisomy 14 above. Moreover, most cases involved a normal female karyotype and maternal cell contamination was not excluded with molecular testing [9]. Whether a false negative uncultured MC truly exists is therefore questionable.

The incidence of CPM involving a copy number variation (CNV) of a single gene, as described by Sabbagh et al. [10], is unknown, and so far only a few cases have been described. But since these mosaic CNVs originate post-zygotically during early embryonic development, it is to be expected that the timing of the event and the distribution of the CNV-containing cells over the different compartments of trophectoderm and inner cell mass will ultimately determine the type of chromosomal mosaicism and clinical relevance, comparable to what is seen for aneuploidies. There is no reason to believe this will be different. Therefore, also in the molecular era it still seems to be best practice to analyse DNA from the uncultured MC and CTB separately instead of analysing a mixture of both cell lineages, to allow for proper and accurate interpretation of the results and minimize the risk for a secondary invasive test, which unnecessarily delays a definitive prenatal diagnosis.
